# A Question of Trust: Does Mistrust or Perceived Discrimination Account for Race Disparities in Advance Directive Completion?

**DOI:** 10.1093/geroni/igx017

**Published:** 2017-09-07

**Authors:** Catheryn S Koss, Tamara A Baker

**Affiliations:** 1 Gerontology Program, California State University, Sacramento.; 2 Department of Psychology, University of Kansas, Lawrence.

**Keywords:** Advance care planning, Decision making, Disparities, Minority issues, Race

## Abstract

**Background and Objectives:**

Advance directive completion is associated with end-of-life quality indicators such as dying at home and receiving hospice care. Black older adults are less likely to complete advance directives than their white counterparts. The underlying reasons for these race disparities are not well understood.

**Research Design and Methods:**

In two related studies, data from the Health and Retirement Study were used to examine whether mistrust in health care providers and/or perceived discrimination accounted for lower rates of advance directive completion by black older adults in the United States. Odds of advance directive completion were modeled using logistic regression and multiple measures of trust in health care providers and both medical and nonmedical perceived discriminatory treatment.

**Results:**

In Study 1 (*n* = 699), controlling for medical mistrust did not reduce the gap between black and white participants’ odds of possessing advance directives. In Study 2 (*n* = 2,736), higher percentages of black participants reported experiencing medical and nonmedical discriminatory treatment. However, none of the measures of discrimination accounted for black participants’ lower odds of possessing advance directives.

**Discussion and Implications:**

These results call into question the common assertion that mistrust in medical providers or the health care system contributes to lower rates of advance care planning by black older adults. Future research should examine the potential relationships between advance directive completion and other dimensions of discrimination.

**Translational Significance**

The results suggest that trust in medical providers is high among both white and black older adults. Health care providers are therefore well positioned to counsel older patients about advance care planning. In addition, the results raise doubts about perceived discrimination and mistrust as underlying causes of race disparities in advance directive completion, suggesting that other potential explanatory factors should be examined.

## Background and Objectives

Approximately 40% of older adults in the United States become unable to make medical decisions at the end of life (Silveira, Kim, & Langa, 2010). Advance care planning (ACP) is an ongoing process of contemplating, discussing, and documenting instructions about how and by whom health care decisions should be made in the event of incapacity (Sudore et al., 2008). ACP often results in a written advance directive usually consisting of two parts: a living will and a durable power of attorney for health care (Institute of Medicine, 2015). The living will is used to state what medical treatments one would or would not wish to receive under certain conditions, such as persistent unconsciousness or late-stage dementia. The power of attorney names one or more persons (called an attorney-in-fact) who will be legally authorized to carry out these wishes and make medical decisions, if necessary, on behalf of the incapacitated person.

Advance directive completion is associated with important end-of-life quality indicators, including dying at home rather than in a hospital as well as receiving hospice care earlier and longer (Bischoff, Sudore, Miao, Boscardin, & Smith, 2013; Teno, Gruneir, Schwartz, Nanda, & Wetle, 2007). Given these benefits, considerable effort is made in the United States to encourage ACP. For example, the *Patient Self-Determination Act* requires all hospitals, long-term care facilities, hospices, home health agencies, and health maintenance organizations that receive Medicare or Medicaid funding to provide information about advance directives at admission or enrollment (Institute of Medicine, 2015).

Approximately half of U.S. adults aged 65 and older report having completed a living will or advance directive (Pew Research Center, 2009; Rao, Anderson, Lin, & Laux, 2014). Numerous studies have observed that, compared to whites, black older adults are significantly less likely to engage in ACP even after controlling for other sociodemographic characteristics (Gerst & Burr, 2008; Koss & Baker, 2017; Rao et al., 2014). This disparity is particularly concerning given the relationship between ACP and end-of-life care. Black elders are less likely than whites to receive hospice or other palliative care and to have their pain effectively managed at the end of life (Bullock, McGraw, Blank, & Bradley, 2005; Crawley et al., 2000).

Despite numerous studies, the underlying mechanisms for ACP race disparities are still not well understood. Proposed reasons for low rates of advance directive completion among black older adults include greater religiosity, reluctance to acknowledge terminal prognosis, more common preference for life-sustaining treatment, and lower levels of health literacy (Ladd, 2014; Sanders, Robinson, & Block, 2016). One frequently-asserted explanation for race disparities is mistrust in health care providers or the medical system (Sanders et al., 2016). Across numerous studies, blacks report lower levels of trust in the health care system than whites (Armstrong et al., 2008; Carr, 2011; Krakauer, Crenner, & Fox, 2002). This lack of trust is rooted in centuries of mistreatment during which black patients were subjected to medical experimentation without their knowledge or permission, discriminated against by providers and institutions, and denied access to quality care (Feagin & Bennefield, 2014). Even though ACP is designed to improve care by ensuring that patients’ wishes are honored, some black older adults may be reluctant to document their end-of-life wishes out of fear that their instructions may be misinterpreted or misused (Carr, 2011; Waters, 2001).

Concerns about mistreatment or undertreatment are frequently mentioned as reasons not to engage in ACP by participants in qualitative studies (Blackhall et al., 1999; Bullock, 2006; Bullock et al., 2005; Daaleman & Emmett, 2008; Periyakoil, Neri, & Kraemer, 2015; Rhodes, Batchelor, Lee, & Halm, 2015; Waters, 2001). However, the few quantitative studies that have been conducted have not found that medical mistrust accounts for race disparities in ACP (Carr, 2011; Ejaz, 2000; Huang, Neuhaus, & Chiong, 2016; Ko & Lee, 2014). One study observed that controlling for health care system mistrust narrowed the gap between the odds of having an advance directive for white and black older adults, but whites remained almost three times as likely to engage in written ACP (Johnson, Kuchibhatla, & Tulsky, 2008). These studies have all relied on small, nonrepresentative samples and/or single measures of medical mistrust.

Although perceived health care discrimination may be particularly detrimental to ACP by directly undermining the patient-provider relationship, experiencing discriminatory treatment outside of the health care system—such as in banking or employment—may also result in lower levels of trust generally and contribute to race disparities in advance directive completion (Armstrong et al., 2013). To our knowledge, no study has yet tested the association between perceived discriminatory treatment and ACP or determined whether perceived discrimination accounts for lower rates of advance directive completion by black older adults.

Perceived discrimination may be measured as ongoing discriminatory treatment or negative life events (Thrasher, Clay, Ford, & Stewart, 2012). For example, The *Everyday Discrimination Scale* measures chronic experiences of unfair treatment in daily life (Williams & Mohammed, 2009), whereas the *Experiences of Discrimination Scale* captures discrete incidents of discriminatory treatment over the life course (Thrasher et al., 2012). Black individuals report experiencing both everyday and lifetime discrimination at higher rates than whites (Williams, Yu, Jackson, & Anderson, 1997).

Both everyday and lifetime discrimination may negatively impact advance directive completion. Perceived everyday discrimination is associated with lower health care service utilization such as routine physical exams and other preventive services (Benjamins, 2012). Those who regularly interact with health care providers are more likely to complete advance directives (Rao et al., 2014). Experiencing day-to-day discriminatory treatment has also been found to decrease agreeableness and conscientiousness in older adults (Sutin, Stephan, & Terracciano, 2016), personality traits which have been linked to willingness to engage in ACP (Carr, 2012; Ha & Pai, 2012). Major discriminatory events may impact health behaviors, including ACP, by undermining trust in health and other institutions as well as contributing to the accumulation of disadvantages in informational, financial, and social resources that leads to health disparities (Phelan, Link, & Tehranifar, 2010).

In two related studies, we examined whether mistrust in health care providers, perceived everyday discrimination (nonmedical and medical), and/or perceived lifetime discrimination (nonmedical and medical) accounted for disparities in advance directive completion between white and black older adults. If race disparities were attributable to mistrust in health care providers and/or discriminatory treatment, one would expect the data to display three trends. First, black older adults should report higher levels of mistrust and/or discriminatory treatment than their white counterparts. Second, mistrust and/or perceived discrimination should be negatively associated with advance directive completion. Third, the gap between the odds of possessing an advance directive for white and black participants should narrow once mistrust and/or perceived discrimination measures are included in the models.

## Research Design and Method

### Data and Samples

Research was conducted using data from the 2012 wave of the Health and Retirement Study (HRS, 2012), a longitudinal, nationally representative survey of older adults in the United States sponsored by the National Institute on Aging (Grant NIA U01AG009740) and administered by the University of Michigan. Analyses were limited to data from participants who were either non-Hispanic black or non-Hispanic white, aged 65 and older, and residing in the 50 United States. One person was randomly selected from each household with more than one eligible participant.

The 2012 wave of the HRS provided a unique opportunity to quantitatively examine for the first time whether mistrust of health care providers, medical or nonmedical everyday discrimination, and/or experiences of medical or nonmedical lifetime discriminatory events account for race disparities in advance directive completion. Two new questions about living wills and durable powers of attorney for health care were added to the 2012 core HRS interview and asked of all participants 65 and older. The 2012 wave also included an experimental module administered to a randomly selected subset of participants that contained questions about stereotyping and unfair treatment by doctors and other health care providers. These data (*n* = 699) were used to test to what extent lower rates of advance directive completion among older blacks could be explained by mistrust in health care providers (Study 1). In addition, a randomly selected 50% of HRS participants were asked about perceived everyday and lifetime discrimination in the supplemental Psychosocial and Lifestyle Questionnaire (Smith et al., 2013). These data (*n* = 2,736) were used to test to what extent lower rates of advance directive completion among older blacks could be explained by higher perceived everyday and/or lifetime discrimination (Study 2).

### Measures

#### Advance directive completion

The outcome variable in both Study 1 and Study 2 was whether or not a person had an advance directive, measured with two yes/no questions: (1) “Have you provided written instructions about the care or medical treatment that you want to receive if you can not make those decisions yourself? This is sometimes called a ‘Living Will.’” and (2) “Have you made any legal arrangements for a specific person or persons to make decisions about your care or medical treatment if you can not make those decisions yourself? This is sometimes called a ‘Durable Power of Attorney for Health Care.’” Participants who responded positively to either question were coded as having an advance directive. Those who responded negatively to both questions were coded as not possessing an advance directive (reference group).

#### Race

Race was defined in both studies as either non-Hispanic white or non-Hispanic black (reference group).

#### Covariates

Both Study 1 and Study 2 included the same demographic and health covariates: gender, age in years, education (*less than high school, high school/general educational development test [GED], some college, bachelor’s degree or higher*), marital status (*married/partnered, widowed, separated/divorced, never married*), self-rated health (a five-point scale ranging from *poor* to *excellent*), a composite dichotomous variable indicating if the person had experienced hospitalization, outpatient surgery, and/or nursing home admission in the prior 2 years (yes/no), whether the individual has a regular health care provider other than the emergency room (yes/no), total annual household income (U.S. dollars, log-transformed), and total household net wealth (U.S. dollars, log-transformed). Each has been found in prior research to be associated with ACP (Institute of Medicine, 2015; Pew Research Center, 2009; Rao et al., 2014). In addition, body mass index (BMI; weight in kilograms divided by height in meters squared) and current smoker status (yes/no) were controlled because these may contribute to feelings of being judged or discriminated against by medical professionals or others.

#### Study 1 explanatory variables

Two variables measuring trust in doctors and other health care providers were included.

##### Worry about being judged

Participants were asked whether, when they visited the doctor, they worried that the doctor or medical staff judged them because of their race, ethnicity, gender, age, weight, religion, financial situation, or some other reason. Those who responded yes for any reason were coded as worrying about being judged by health care providers. Those who answered no to all questions were coded as not being worried about being judged (reference group).

##### Trust doctor’s judgment

Individuals were asked (yes/no) if they agreed with the statement, “When you visit the doctor, you completely trust the doctor’s judgment about your medical care.”

#### Study 2 explanatory variables

Two perceived discrimination scales—one addressing everyday discriminatory treatment and one related to lifetime discriminatory events—were administered through the supplemental psychosocial questionnaire. Four composite variables were constructed from these data to measure nonmedical everyday discrimination, medical everyday discrimination, nonmedical lifetime discrimination, and medical lifetime discrimination.

##### Nonmedical everyday discrimination

Participants were asked how frequently (*almost everyday*, *at least once a week*, *a few times a month*, *a few times a year*, *less than once a year*, *never*) they experienced the following types of discriminatory treatment in day-to-day life: (1) treated with less courtesy or respect than others, (2) receive poorer service than others at restaurants or stores, (3) people act as if they think you are not smart, (4) people act as if they are afraid of you, and (5) threatened or harassed. Those who reported never experiencing any of these forms of everyday discrimination were categorized as “never” (reference group). Those who reported experiencing any form of discrimination but no more than once per year were classified as “rarely.” Participants who reported experiencing any discriminatory treatment at least a few times per year were categorized as “more than rarely” (Benjamins, 2012).

##### Medical everyday discrimination

Participants were asked how often (*almost everyday*, *at least once a week*, *a few times a month*, *a few times a year*, *less than once a year*, *never*) they “receive poorer service or treatment than other people from doctors or hospitals.” Those who reported ever receiving poorer service were coded 1 indicating they had experienced some form of everyday medical discrimination.

##### Nonmedical lifetime discrimination

Participants were asked (yes/no) whether they had at any point in their lives been: (1) unfairly dismissed from a job; (2) unfairly not been hired for a job; (3) unfairly denied a promotion; (4) unfairly prevented from moving into a neighborhood because the landlord or realtor refused to sell or rent; (5) unfairly denied a bank loan; or (6) unfairly stopped, searched, questioned, physically threatened, or abused by the police. Those who responded affirmatively to one or more of these questions were coded 1 indicating they had experienced some form of nonmedical lifetime discrimination.

##### Medical lifetime discrimination

The dichotomous variable of medical lifetime discrimination was based on participants’ reports of ever being “unfairly denied health care or treatment” (yes/no).

### Analyses

For each study, descriptive statistics were tabulated for the entire sample and stratified by race. Logistic regression was used to estimate unadjusted and adjusted odds ratios (ORs) predicting advance directive completion. Data were prepared for analysis and descriptive statistics were generated in STATA (version 14; StataCorp LP, College Station, TX). Logistic regressions were run in Mplus (version 7.4; Muthén & Muthén, Los Angeles, CA) with maximum likelihood estimation with robust standard errors. Missing data were handled with full information maximum likelihood estimation.

Because Study 1 used data from an experimental module administered to a relatively small subsample, weights were not applied. Weighted data were used in Study 2 to account for the sampling design of the HRS, including purposeful oversampling of black participants. When weights are applied, the HRS data are representative of the noninstitutionalized U.S. population older than the age of 50 (Sonnega et al., 2014).

### Ethical Considerations

This research was exempted from review by the University of Kansas institutional review board.

## Results

### Study 1: Trust in Medical Providers

#### Descriptives

Unweighted descriptive statistics are presented in Table 1. Rates of advance directive completion were higher among the white participants, with 64% possessing advance directives compared to 43% of blacks (*p* < .001). A slightly higher percentage of white participants expressed worry about being judged by health care providers (18%) compared to black participants (15%), but this difference was not statistically significant. Eighty percent of both groups agreed with the statement that they completely trusted their doctors’ judgment about their care. The white participants were on average slightly older, more educated, more likely to be married, less likely to be divorced or widowed, healthier, had lower BMI, and had substantially higher household incomes and net wealth. They were also more likely than black participants to have been hospitalized, undergone outpatient surgery, and/or gone into a nursing home in the past 2 years as well as to have a regular health care provider. There was no statistically significant difference in smoking rates.

**Table 1.** Sample Characteristics, Study 1 (Unweighted) and Study 2 (Weighted)

Variable	Study 1			Study 2		
	Total (*n* = 699)	Black (*n* = 103)	White (*n* = 596)	Total (*n* = 2,736)	Black (*n* = 404)	White (*n* = 2,332)
Female (%)	58	63	57	59	62	59
Age (M)	75.2	73.5	75.5	74.8	73.3	75.0
Education (%)						
<High school	14	30	11	17	38	15
High school/GED	39	37	40	36	32	37
Some college	24	18	25	24	18	24
College or above	23	15	25	23	11	24
Marital status (%)						
Married	62	45	65	48	31	50
Divorced	9	17	8	15	25	14
Widowed	28	36	26	32	32	32
Never married	1	3	1	5	12	4
Self-rated health (%)						
Excellent	7	5	8	8	5	9
Very good	31	24	32	33	21	34
Good	36	34	36	33	34	32
Fair	19	27	17	19	27	18
Poor	7	10	6	7	13	7
Hospital/surgery/nursing home in past 2 years (%)	43	34	45	45	42	46
Regular health care provider (%)	89	79	90	88	81	89
Body mass index (M)	28.1	29.4	27.9	27.9	29.5	27.7
Current smoker (%)	8	10	8	10	11	9
HH income (Median) (US$)	32,198	18,360	34,572	29,464	18,015	31,313
Net HH wealth (Median) (US$)	212,000	75,000	261,300	201,350	49,306	237,700
Worry about medical staff judgments (%)	18	15	18	—	—	—
Completely trust doctor’s judgment (%)	80	80	80	—	—	—
ED non-med discrimination (%)						
Never	—	—	—	45	38	46
Rarely	—	—	—	19	13	20
>Rarely	—	—	—	35	49	34
LT non-med discrimination (%)	—	—	—	24	28	24
ED med discrimination (%)	—	—	—	14	19	14
LT med discrimination (%)	—	—	—	2	3	2
Advance directive (%)	61	43	64	58	34	61

*Note:* ED = everyday; HH = household; LT = lifetime.

#### Bivariate analyses

The unadjusted odds of having an advance directive were more than two times as high for white participants compared to black participants (OR = 2.37, *p* < .001). Age, education, widowhood, being female, log net wealth, hospitalization, outpatient surgery and/or nursing home stay in the past 2 years, lower BMI, and being a nonsmoker were each associated with higher odds of advance directive completion. Worrying about medical staff making judgments about oneself was marginally associated with higher odds of having an advance directive (OR = 1.43, *p* < .10). Whether an individual completely trusted his or her doctor’s judgment was not significantly associated with advance directive completion.

#### Multivariable analyses

Results from multivariable analyses are reported in Table 2. First, a base model was estimated calculating the OR for advance directive completion between white and black participants after controlling for gender, age, education, marital status, self-rated health, hospitalization, outpatient surgery, and/or nursing home admission in the prior 2 years, BMI, current smoker, regular health care provider, total annual household income (log), and total household net wealth (log). Compared to the bivariate model, the difference in the odds of advance directive completion for whites and blacks narrowed once the demographic and health covariates were included, but white participants were still almost twice as likely to possess advance directives (OR = 1.79, *p* < .05).

**Table 2.** Study 1, Logistic Regression Models Measuring Associations Between Advance Directive Completion and Trust in Health Care Providers (*n* = 699)

Variable	Base model, OR [95% CI]	Separate models, OR [95% CI]		Combined model, OR [95% CI]
White (ref = black)	1.79* [1.17, 2.73]	1.77* [1.16, 2.70]	1.78* [1.17, 2.73]	1.76* [1.15, 2.69]
Worry judged	—	1.44 [0.98, 2.11]	—	1.50^†^ [1.01, 2.23]
Trust doctor’s judgment	—	—	1.15 [0.82, 1.62]	1.23 [0.87, 1.74]

*R* ^2^	.14	.15	.15	.15

*Note:* OR = odds ratio; CI = confidence interval; worry judged = when visiting the doctor, worry that the doctor or medical staff judged them because of their race, ethnicity, gender, age, weight, religion, financial situation, or some other reason. Odds ratios adjusted for age, gender, education, marital status, self-rated health, hospitalization, outpatient surgery, and/or nursing home stay in prior 2 years, regular health care provider, body mass index, current smoker, log household income, and log household net wealth.

^†^ *p <* .10; *p < .05.

To examine the relationships between each measure of mistrust in health care providers and advance directive completion before and after adjusting for the other mistrust variable, each was entered into two separate logistic regression models (separate models) and then together in the same model (combined model) along with race and covariates. Neither measure of trust in medical providers was significantly associated with advance directive completion when entered separately. In the combined model, being worried about being judged was positively associated with advance directive completion (OR = 1.50), but only at the *p* <.10 level. Including the two trust variables did not substantially change the OR of advance directive completion between white and black participants (OR = 1.76, *p <* .05).

### Study 2: Perceived Discrimination

#### Descriptives

Weighted descriptive statistics are presented in Table 1. Rates of advance directive completion were higher among white participants (*p* < .001), with 61% of whites possessing advance directives compared to 34% of black participants. Almost half (49%) of the black participants reported experiencing nonmedical discriminatory treatment at least a few times a year compared to 34% of whites, whereas 46% of white participants reported no everyday discriminatory treatment compared to 38% of blacks (*p* = .04). The race difference was less pronounced for lifetime nonmedical discrimination, with 28% of black and 24% of white participants reporting one or more discriminatory incidents during their lives (*p* = .08). Perceived everyday medical discrimination was more common among black participants (19%) than whites (14%; *p* = .01), but reports of lifetime medical discrimination were rare for both groups (3% of blacks and 2% of whites) and not statistically different. Compared to black participants, white participants were on average slightly older, had completed more years of education, were more likely to be married, were healthier, had lower BMI, were more likely to have regular health care providers, and had substantially higher household incomes and net wealth. There were no statistically significant differences in hospitalization, outpatient surgery, and/or nursing home admission or being a current smoker.

#### Bivariate analyses

The unadjusted odds of having an advance directive were more than three times as high for whites than blacks (OR = 3.12, *p* < .001). Older age, higher education, widowhood, greater income, greater net wealth, having been hospitalized, undergone outpatient surgery and/or admitted to a nursing home, lower BMI, being a nonsmoker, and having a regular health care provider were each separately associated with higher odds of advance directive completion. Three of the four perceived discrimination measures displayed negative relationships with advance directive completion: everyday nonmedical discrimination rarely (OR = .78, *p* < .05) or more than rarely (OR = .80, *p* < .05), everyday medical discrimination (OR = .79, *p* < .05), and nonmedical lifetime discrimination (OR = .83, *p* < .05). There was no significant relationship between advance directive completion and lifetime medical discrimination.

#### Multivariable analyses

Results from multivariable analyses are reported in Table 3. First, a base model was estimated calculating the OR for advance directive completion between white and black participants after controlling for gender, age, education, marital status, self-rated health status, hospitalization, outpatient surgery, and/or nursing home admission in the prior 2 years, BMI, current smoker, regular health care provider, total annual household income (log), and total household net wealth (log). Compared to the bivariate model, the gap between the odds of having an advance directive for white and black participants narrowed once the health and demographic variables were controlled, but whites were twice as likely to have completed advance directives (OR = 2.00, *p* < .001).

**Table 3.** Study 2, Logistic Regression Models Measuring Associations Between Advance Directive Completion and Perceived Discrimination (*n* = 2,736)

Variable	Base model, OR [95% CI]	Separate models, OR [95% CI]				Combined model, OR [95% CI]
White (ref = black)	2.00*** [1.61, 2.47]	2.03*** [1.63, 2.53]	2.00*** [1.62, 2.47]	2.00*** [1.61, 2.47]	2.00*** [1.61, 2.48]	2.04*** [1.64, 2.54]
ED non-med (ref = never)						
Rarely		0.77* [0.63, 0.94]	—	—	—	0.79^†^ [0.64, 0.97]
>Rarely		1.01 [0.82, 1.25]	—	—	—	1.06 [0.83, 1.36]
LT non-med		—	1.05 [0.85, 1.31]	—	—	1.06 [0.85, 1.33]
ED med		—	—	0.85 [0.71, 1.03]	—	0.81 [0.64, 1.03]
LT med		—	—	—	1.16 [0.56, 2.40]	1.21 [0.56, 2.63]

*R* ^2^	.17	.18	.18	.18	.18	.18

*Note:* CI = confidence interval; ED med = everyday medical discrimination; ED non-med = everyday nonmedical discrimination; LT med = lifetime medical discrimination; LT non-med = lifetime nonmedical discrimination; OR = odds ratio. Odds ratios adjusted for age, gender, education, marital status, self-rated health, hospitalization, outpatient surgery, and/or nursing home stay in past 2 years, regular health care provider, body mass index, current smoker, log household income, and log household net wealth.

^†^ *p <* .10; *p < .05; ***p < .001.

To examine the relationships between each perceived discrimination measure and advance directive completion before adjusting for the other three discrimination variables, each was entered into a separate logistic regression model along with race and covariates (separate models). Those who experienced nonmedical everyday discriminatory treatment rarely were less likely to have advance directives compared to those who never experienced everyday discrimination (OR = .77, *p <* .05). None of the other perceived discrimination measures were significantly associated with advance directive completion in separate models.

When all four perceived discrimination variables were entered simultaneously (combined model), the OR for never versus rare nonmedical everyday discrimination became only marginally significant (OR = .79, *p <* .10). The associations between advance directive completion and the other discrimination measures continued to be nonsignificant. After adding the four discriminatory treatment measures to the model, the OR for possessing an advance directive between white and black older adults was substantially unchanged compared to the base model (OR = 2.04, *p <* .001). Whites continued to be twice as likely to complete advance directives.

## Discussion and Implications

Mistrust resulting from a long history of discriminatory treatment is often proposed in the literature as a potential explanatory factor for race disparities in advance directive completion. Two related studies used quantitative data to test, for the first time, whether lower rates of advance directive completion by older blacks could be explained by one or more measures of mistrust in health care providers and/or perceived discriminatory treatment.

Results from Study 1 fail to support the proposition that lower levels of trust in medical providers contribute to disparities in advance directive completion between white and black older adults. Approximately the same proportions of black and white participants reported worrying about being judged by health care providers and trusting doctors’ judgment. Neither measure was associated with advance directive completion, nor did either account for black participants’ lower odds of possessing advance directives. These findings are largely consistent with the few previous studies that have tested the relationship between medical mistrust and ACP (Carr, 2011; Ejaz, 2000; Huang et al., 2016; Johnson et al., 2008; Ko & Lee, 2014).

Similarly, the results from Study 2 do not support the proposition that lower rates of advance directive completion by black older adults are attributable to perceived discriminatory treatment. As anticipated, a greater proportion of black participants reported experiencing both nonmedical and medical discrimination. Those who reported rarely experiencing nonmedical everyday discrimination were somewhat less likely to have advance directives compared to those who never experienced discriminatory treatment. However, none of the other measures of perceived discrimination were significantly associated with advance directive possession in multivariable analyses. Furthermore, perceived discrimination did not account for substantially lower odds of advance directive completion for black participants compared to whites.

Post-hoc sensitivity analysis testing whether effects differed for living will and durable power of attorney for health care completion resulted in a similar finding that neither discrimination nor mistrust accounted for race disparities in either type of written ACP. Race disparities were greater for living will completion than for durable powers of attorney. Trust in one’s doctor and experiencing rare nonmedical discrimination were somewhat more predictive of appointing an attorney-in-fact than completing a living will, although the relationships were only marginally significant at *p* <.10 in combined models.

Despite the fact that race disparities in advance directive completion remain poorly understood, these data offer some positive evidence. Perceived medical discrimination was relatively uncommon and trust in health providers was high. Furthermore, perceived discrimination and mistrust in the health care system do not appear to be significant barriers to advance directive completion. These findings should hearten advocates promoting end-of-life planning, particularly in black communities where perceived discrimination is likely to be more prevalent.

Several limitations should be acknowledged. Data were self-reported, so it is possible that some participants were mistaken about whether or not they had completed advance directives. Self-reporting of discriminatory treatment is also problematic due to the tendency to underreport (Armstrong et al., 2013). There were no data about when ACP took place, so conclusions about causality cannot be reached. The timing of discriminatory treatment may also matter, so it would be beneficial in future research to explore how remote and recent experiences of discrimination as well as discrimination at more or less formative stages in life may be influential on ACP (Gee, Walsemann, & Brondolo, 2012).

The measurements used in these two studies and in previous research may not capture how medical mistreatment or other forms of discrimination impact ACP. Data were limited to personally experienced discriminatory treatment and trust in one’s own medical providers. Many black older adults have been required to develop coping mechanisms that may allow them to either ignore or otherwise handle discriminatory aggressions in a manner that does not result in hindering their own decisions to engage in ACP or not. However, how perceptions of general discrimination may contribute to ACP disparities has yet to be fully explored.

Perceptions of institutional or systemic racial discrimination, as measured by questions about unfair treatment of blacks generally (LaVeist, Nickerson, & Bowie, 2000) could influence willingness to complete advance directives regardless of whether one has personally experienced discriminatory treatment. Recent research suggests that witnessing discriminatory treatment of others can be just as detrimental to health as experiencing discrimination directly (Quinlan et al., 2016). It would be worth exploring whether “secondhand” discrimination, such as witnessing discriminatory medical treatment of a parent or spouse, may play a role in decisions to engage in ACP.

Interactions between patients and health care providers may be shaped by structural discrimination and contribute to lower rates of advance directive completion by black older adults. Physicians tend to spend clinical time differently and employ different communication styles depending on whether the patient is white or black (Johnson, Roter, Powe, & Cooper, 2004; Oliver, Goodwin, Gotler, Gregory, & Stange, 2001). The “racial framing” of health care services provided by a predominantly white medical profession may lead to systemic race disparities in how ACP information and assistance are offered based on implicit assumptions about patients’ receptivity or abilities (Feagin & Bennefield, 2014). These biases and accompanying differential treatment may be unrecognized by both patients and providers.

Although black participants were more likely to report experiencing different forms of discriminatory treatment, neither perceived discrimination nor mistrust in health care providers explained race differences in advance directive completion. These results call into question the common assertion that mistrust accounts for lower rates of ACP by older blacks. Future research should examine the potential relationships between ACP and other dimensions of discrimination.

We still do not fully understand the barriers that contribute to lower rates of ACP by black older adults as well as other diverse race populations. Finding answers will require a careful examination of the historical context of health, health outcomes, access to resources, and acceptability of treatment among these groups. Advancing the literature in this area allows for a more thorough exploration of the many social, behavior, and historical factors that facilitate and/or hinder health behaviors, including ACP.

## Funding

This work was supported by the Borchard Foundation Center on Law and Aging.

## Conflicts of Interest

None reported.
